# Antioxidant Defense in Primary Murine Lung Cells following Short- and Long-Term Exposure to Plastic Particles

**DOI:** 10.3390/antiox12020227

**Published:** 2023-01-18

**Authors:** Anke Schmidt, Melissa Mühl, Walison Augusto da Silva Brito, Debora Singer, Sander Bekeschus

**Affiliations:** 1 ZIK *Plasmatis*, Leibniz Institute for Plasma Science and Technology (INP), Felix-Hausdorff-Str. 2, 17489 Greifswald, Germany; 2 Department of General Pathology, State University of Londrina, Rodovia Celso Garcia Cid, Londrina 86020-000, Brazil; 3 Department of Dermatology and Venerology, Rostock University Medical Center, Strempelstr. 13, 18057 Rostock, Germany

**Keywords:** environment, inflammation, oxidative stress, plastic particles, toxicity, uptake

## Abstract

Polystyrene nano- and micro-sized plastic particles (NMP) are one of the common plastic materials produced that dramatically pollute the environment, water, and oceanic habitats worldwide. NMP are continuously absorbed by the body through a number of routes, especially via intestinal ingestion, dermal uptake, and inhalation into the lung. Several studies provided evidence of NMP provoking oxidative stress and affecting cellular responses. Yet, the NMP effects on primary lung cells have not been studied. To this end, we isolated and cultured murine lung cells and exposed them short-term or long-term to polystyrene 0.2–6.0 µm-sized NMP. We studied cellular consequences regarding oxidative stress, morphology, and secretion profiling. Visualization, distribution, and expression analyses confirmed lung cells accumulating NMP and showed several significant correlations with particle size. Moreover, we found substantial evidence of biological consequences of small-scale NMP uptake in lung cells. Besides alterations of cytokine secretion profiles resulting in inflammatory responses, indicators of oxidative stress were identified that were accompanied by Nrf2 and β-catenin signaling changes. Our results serve as an important basis to point out the potential hazards of plastic contaminations and uptake in lung cells.

## 1. Introduction

Plastic diversity and versatility make its application indispensable across all areas of everyday life. This, of course, goes hand in hand with its ever-increasing role as a pollutant in aquatic systems and air, recognizing plastic as a serious global environmental concern [1,2]. Emerging airborne pollutants include, for instance, polystyrene, polyvinyl chloride, polyethylene, polycarbonate, polypropylene, polyurethane, and polyethylene terephthalate [3,4]. They imply a high risk to the human respiratory system [5]. Each year, tons of micro- and nanoparticles (NMP) are released into the atmosphere worldwide when NMP-containing water evaporates, or any plastic object is damaged and scraped. Through the air and abrasion from car tires or clothing, such NMP can be inhaled by humans and animals [6,7]. A previous study found 575 to 1008 NMP per square meter of air in every sample collected twice a week for a month [8].

Due to their small size and strong biological penetration, NMP are easily ingested by organisms, causing severe damage to biological functions on a single-cell level [9,10]. Additionally, NMP can release chemical additives into the body from surface-coated organic molecules (protein corona) [11,12]. In humans, plastic uptake, accumulation, and distribution in cells and tissues and the subsequent biomedical relevance are still largely unexplored [13]. Several mechanisms of penetration and adsorption are currently being suggested as possible ways of interaction and entry of NMP into cells and tissue [14] with severe side effects and consequences [15,16]. A variety of NMP exposure routes could adversely affect human health [6,17]. In addition to small particles, particles up to ten micrometers can penetrate all tissues and cells and accumulate in organs causing modifications of physiological processes [18]. Several studies focused on the absorption of polymeric particles via the gastrointestinal tract, with intestinal uptake of plastic-contaminated water or food being the predominant route in humans [19,20,21]. Besides the oral and/or dermal uptake, NMP inhalation from ambient air is another significant route [22,23,24,25]. It has been found that exposure to polystyrene (PS) NMP causes oxidative stress in human lung cells in vitro [26]. Therefore, the accumulation of plastic in the respiratory mucosa and lung tissue is now the focus of environmental research.

In our study, the mechanisms and effects of the accumulation of fluorescence-labeled polystyrene NMP were monitored in cells freshly isolated from mouse lungs. Polystyrene was selected as it is a commonly produced and used plastic type and one of the primary pollutants found in the environment, which can remain there for a long time [27]. Moreover, the functional consequences of NMP uptake were analyzed with PS-NMP of five different sizes following single or repeated exposure over one or four weeks, respectively. The response to a repeated, long-term NMP uptake is probably more realistic to the kind of accumulation observed in our environment. Critical biological processes, viability, metabolism, toxicity, and pathways were investigated using fluorescence-based high-content imaging techniques, flow cytometry, and gene and protein expression analysis.

## 2. Materials and Methods

### 2.1. Lung Cell Culture

The study was conducted in accordance with the regulations of the local ethics committee of the State of Mecklenburg-Vorpommern (Rostock, Germany; Az.: 7221.3-1-044/16) and the guidelines for the care and use of laboratory animals. It was performed according to the recommendations of Good Laboratory Practice and within the guidelines and regulations of animal care guidelines in SKH1-hr hairless immunocompetent mice (Charles River Laboratories, Sulzfeld, Germany). Primary cells were isolated from the lungs by enzyme-mediated digestion according to the recommendations of a dissociation kit. The cell suspension was homogenized in gentleMACS C tubes using a gentleMACS dissociator for obtaining live cells (Miltenyi Biotec, Bergisch-Gladbach, Germany). To separate individual cells, the suspensions were passed through 40 µm MACS SmartStrainers (Miltenyi Biotec). Lung cells were spun down. The resulting mix of lung cells, including pulmonary alveolar epithelial type I and type II cells, were cultured over a total of four weeks in complete Dulbecco’s Minimum Essential Medium (DMEM, PromoCell, Heidelberg, Germany) supplemented with 10% fetal bovine serum, 1% penicillin/streptomycin, and L-glutamine (Sigma-Aldrich, Taufkrichen, Germany) in a humidified incubator at 37 °C with 5% CO_2_. In our experimental setups, early passages from 1 to 4 were used.

### 2.2. Polymer Particles and Lung Cell Exposure

**Figure 1:**
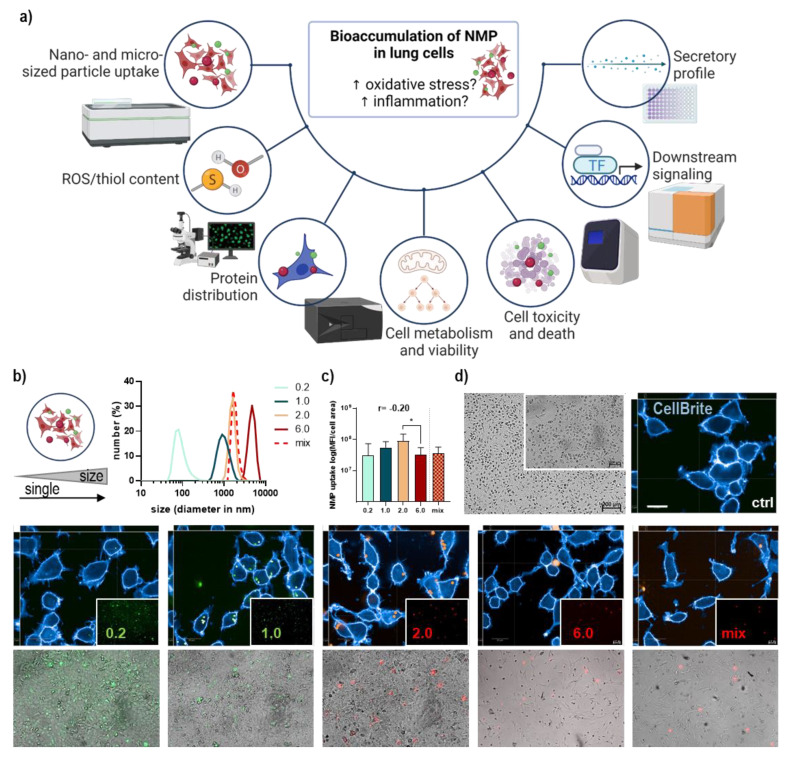
**Methods and algorithm-based uptake analysis of fluorescently labeled NMP in lung epithelial cells**. (**a**) In plastic-exposed cells, several types of analyses were performed, including NMP uptake determination, secretion profiling, gene and protein expression analysis, high-content imaging, and flow cytometry; (**b**) study scheme (right) and size verification via DLS of several polystyrene NMP with fluorescent labeling ranging from 0.2 µm to 6 µm; (**c**) algorithm-based image analysis and calculation of NMP uptake; (**d**) viable lung epithelial cells (mix of primary alveoli and bronchi) cultured in presence of several fluorescent NMP types and maximum intensity projection of representative images (XYZ view) with cell membranes selected in blue and fluorescent particles inside as well as outside of the cell. Scale bars are 5 µm (Z-plane), 50 µm (X and Y planes), and 100–200 µm (brightfield) as indicated. Statistical analysis was done by unpaired, two-tailed *Student*’s *t*-test (*n* > 3) with * *p* < 0.05.

The NMP used in this study were the fluoresbrite (FB) polystyrene microspheres 0.2 (YG, 0.2 µm, #07304), 1.0 (YG, 1 µm, #17154), 2.0 (red, 2 µm, #19508), and 6.0 (red, 6 µm, #19111) (Polyscience, Niles, IL, USA), and polymer microspheres mix (red, 1–5 µm, #FMR-1.3) in aqueous suspension (Cospheric, Goleta, CA, USA). The hydrodynamic diameter was monitored with a dynamic light scattering system (DLS; Zetasizer Ultra, Malvern Panalytics, Kassel, Germany). Stocks were sonicated with a bath sonicator and strongly vortexed before use to avoid aggregation of NMP. Lung cells were incubated with NMP in corresponding cell culture dishes dependent on downstream applications ranging from 6 cm dishes for RNA isolation and protein lysates to 96 well plates for metabolic or viability measurements. Environmentally relevant concentrations of NMP are in the range between 10 and 1000 µg/mL to facilitate studies on biological effects [28,29,30]. In our study, 10× concentrated NMP suspensions were added, reaching 1× the final concentration of 100 µg/mL in PBS. Membrane structures were stained with a blue fluorescent dye (CellBriteBlue; Biotium, Fremont, CA, USA) and visualized along with NMP uptake into the lung cells using a high-content imaging system (Operetta CLS; PerkinElmer, Hamburg, Germany) (Figure 1a). NMP were quantified based on their fluorescence intensity, and the findings were normalized by calculating the ratio of the number of objects per cell area. Image acquisition, data quantification, and validation were performed with the Harmony software (PerkinElmer) analysis package as previously described [30].

### 2.3. Analysis of Intracellular ROS, Thiol Content, Cellular Metabolism, Viability, and Apoptosis

DCF staining was applied to measure intracellular oxidative stress levels. For this, 5 × 10^3^ cells were NMP-treated, incubated for 24 h in a 96-well plate, and washed with PBS. Hydrogen peroxide (H_2_O_2_; final concentration: 100 µM) was used as a positive control. After staining with H_2_-DCF-DA (final concentration: 25 µM; Thermo Fisher Scientific, Dreieich, Germany) at 37 °C for 1 h, fluorescence intensity was assessed, which is associated with the amount of ROS formed. Cells were imaged by fluorescence microscopy (Axio Observer Z.1; Zeiss, Jena, Germany). By flow cytometry, the thiol content was analyzed with a thiol-sensitive reagent (final concentration: 5 µM; Thermo Fisher Scientific), and the granularity of NMP-treated lung cells was determined using the side scatter (SSC) metric and compared to that of untreated cells. NMP-treated lung cells were incubated with resazurin (7-hydroxy-3H-phenoxazin-3-one 10-oxide, final concentration: 100 µM; Alfa Aesar, Kandel, Germany). Resazurin is reduced to fluorescent resorufin by metabolically active cells, and the fluorescence was determined after 2 h using a microplate reader (F200; Tecan, Männedorf, Switzerland) at λ_ex_ 535 nm and λ_em_ 590 nm. Afterward, the viability of lung cells was determined via live–dead analysis. Briefly, cells were stained after NMP exposure with FITC-Annexin V (final concentration: 5 µM; BioLegend, Amsterdam, The Netherlands) along with 4′,6-diamidine-2-phenylindole (DAPI, final concentration: 1 µM; BioLegend) at 37 °C for 30 min. After washing with cold FACS washing buffer, samples were measured using flow cytometry (CytoFLEX LX; Beckman-Coulter, Krefeld, Germany). Data analysis was performed using *Kaluza* analysis software 2.1.3 (Beckman-Coulter).

### 2.4. Quantification of Gene and Protein Expression

After the lysis of cell NMP-treated samples in RNA lysis buffer, total RNA was isolated according to the manufacturer’s instructions (Bio & Sell, Feucht, Germany), and mRNA expression levels were quantified by quantitative real-time PCR (qPCR). Briefly, 1 μg of RNA was transcribed into cDNA, and qPCR was conducted in triplicates using SYBR Green I Master (Roche Diagnostics, Basel, Switzerland). Murine gene-specific primers (Table A1) were used (BioTez, Berlin, Germany). Two housekeeping genes, *GAPDH* and *RPL13A*, were used as an internal normalization control. Gene expression was analyzed using the _∆∆_CT method. For protein analysis, NMP-treated cells were lysed in RIPA buffer containing protease and phosphatase inhibitors (cOmplete Mini, phosSTOP, PMSF; Sigma-Aldrich). The protein expression levels of Nrf2, HO-1, Nqo1, Keap1, Cat, Sod1, β-catenin, and β-actin were determined using corresponding antibodies (Cell Signaling, Heidelberg, Germany) and the WES system together with its Simple Western Software (Protein simple, Santa Clara, CA, USA) according to the manufacturer’s instructions. Gapdh served as housekeeping control. Band intensities were quantified as fold change compared to the untreated control.

### 2.5. Imaging to Study the Bioaccumulation of NMP

Lung cells were seeded on glass coverslips and exposed to NMP after one day. NMP uptake was studied with a live-cell high-content imaging system (Operetta CLS; PerkinElmer), and algorithm-driven image quantification was performed using dedicated imaging software (Harmony 4.9). For immunofluorescence microscopy of samples, cells were fixed in 4% paraformaldehyde (PFA; Sigma-Aldrich) for 20 min and permeabilized with Triton X-100 (0.01% in PBS; Sigma-Aldrich). Fixed cells were incubated with primary antibodies: Nrf2, β-catenin, HO-1 (all Cell Signaling), and FITC- or FR- (flash red) labeled phalloidin for actin cytoskeleton staining (both BioLegend), followed by staining with secondary antibodies conjugated to Alexa Fluor 488 for red NMP or Alex Fluor 594 for green NMP, respectively (both Life Technologies, Ober-Olm, Germany). DAPI was used for nuclear counterstaining, and samples were mounted onto glass slides (VectaShield; Biozol, Hamburg, Germany) prior to fluorescence microscopy using an Axio Observer Z.1 (Zeiss).

### 2.6. Multiplex Chemokine and Cytokine Profiles

Using multiplex cytokine detection technology (LegendPLEX; BioLegend), cytokine profiles were measured in supernatants of lung cells exposed to NMP according to the manufacturer’s instructions. The bead-based sandwich immunoassay was measured using flow cytometry (CytoFLEX S; Beckman-Coulter) targeting tumor necrosis factor-alpha (TNFα), interferon (IFN) α and γ, monocyte chemotactic protein (MCP) 1 (or CCL2) and chemokine (C-X-C motif) ligand (CXCL) 9, and nine interleukins (IL1β, IL6, IL10, IL12p70, IL17A, IL18, IL23, and IL33). Appropriate data analysis software (Vigene Tech, Carlisle, MA, USA) was utilized for target quantification.

### 2.7. Statistical Analysis

Data are presented as mean + SE of at least three independent experiments. Graphing was completed using Prism software 9.5.0 (GraphPad Software, San Diego, CA, USA). Statistical analysis was performed by one-way analyses of variants (ANOVA) for multiple group comparison and *Student*’s *t*-test for comparison between two groups with *p*-values indicated by * *p* < 0.05, ** *p* < 0.01, and *** *p* < 0.001, as in figure legends. All analyses were *Pearson*-correlated against NMP sizes using prism software.

## 3. Results

### 3.1. Lung Epithelial Cells’ NMP Uptake Affected Metabolism, ROS Formation, and Viability

**Figure 2:**
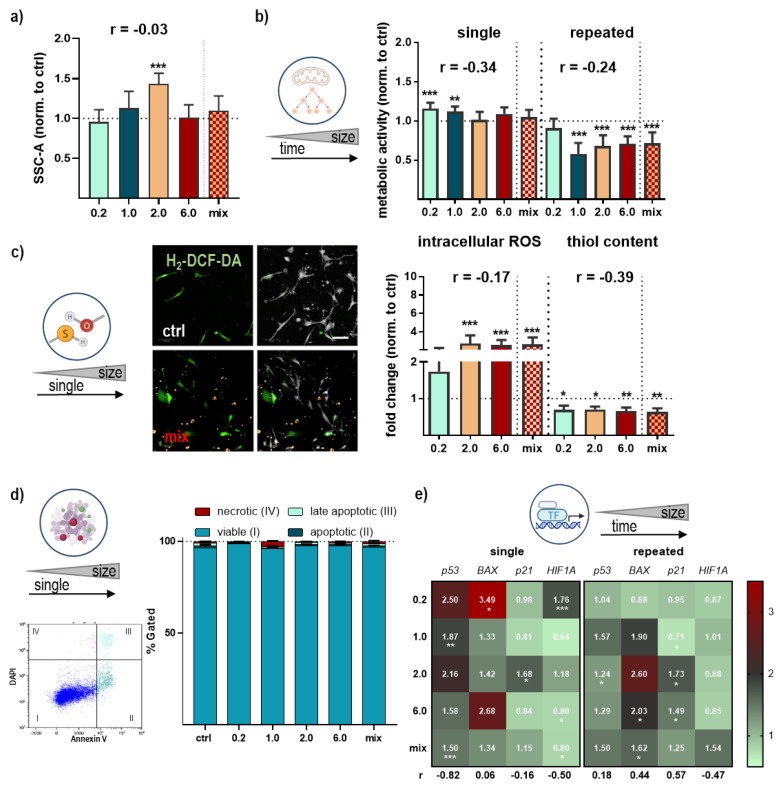
**Cellular metabolism, intracellular ROS, thiol content, viability, and apoptosis in NMP-treated lung cells**. (**a**) Quantification of side-scatter (SCC) signals from individual cells by flow cytometry analysis; (**b**) study scheme and metabolic activity of lung cells exposed to NMP after single or repeated treatment; (**c**) representative images of H_2_-DCF-DA staining in untreated (ctrl) and NMP-treated lung cells (mix) and quantification of ROS by high-content image as well as thiol content analysis using flow cytometry; (**d**) representative FACS density-plot of untreated lung cells; determination of viable and early and late apoptotic and necrotic cells after FITC-Annexin V and DAPI staining using flow cytometry; (**e**) heatmaps of quantification of apoptosis-related *p53* and *BAX* mRNA, cell-cycle-related *p21* mRNA, and *HIF1A* mRNA after single (left) and repeated (right) treatment using qPCR. Data were normalized to *GAPDH*/*RLP13A* and untreated controls (ctrl) and presented as mean + SE. Statistical analysis was done by unpaired, two-tailed *Student*’s *t*-test (*n* > 3) with * *p* < 0.05, ** *p* < 0.01, and *** *p* < 0.001. Scale bar is 50 µm (**c**).

**Figure A1:**
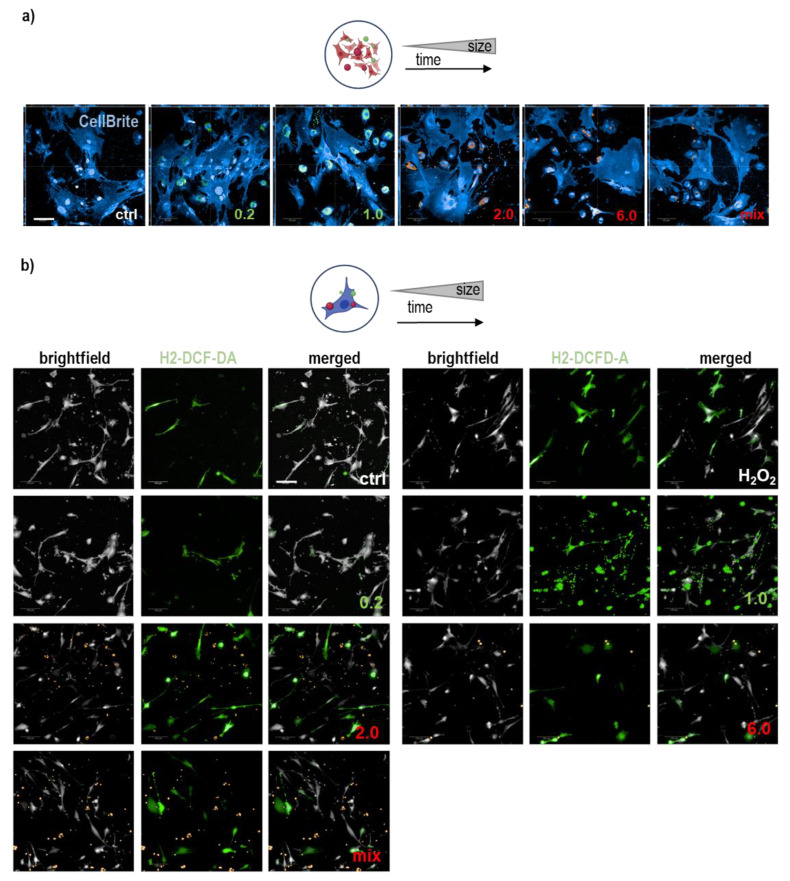
(**a**) Study scheme (top) and maximum intensity projection of representative images (XYZ view) of murine lung cells with cell membranes selected in blue (CellBrite) and fluorescent particles inside as well as outside of the cell in green and red (green for 0.2 and 1 µm, and red for 2.0, 6.0 µm, and mix); (**b**) representative images of H_2_-DCF-DA staining (green) in untreated (ctrl) and NMP-treated lung cells with NMP ranging from 0.2 to 6 µm and a mixture between 1 and 5 µm. Scale bars are 5 µm (Z-plane, **a**) and 50 µm (X and Y planes, **a**,**b**).

Lung epithelial cells are part of the first line of defense in the lung and are involved in initiating and modulating immune responses in case of foreign particles or substances infiltrating the lung. Therefore, we isolated a mix of subpopulations of lung cells, including pulmonary alveolar and bronchial epithelial cells, and exposed them to NMP once (for one week) or repeatedly for four weeks. Uptake, distribution, toxicity, metabolic activity, secretory pattern, and changes in downstream signaling were observed and analyzed using a broad spectrum of methods (Figure 1a). Green or red fluorescently labeled spheric polystyrene NMP were utilized in a sizes range between 0.2 µm, 1.0 µm, 2.0 µm, and 6.0 µm. Furthermore, we added a mixture of around 1–5 µm sized particles. All types of particles were validated using dynamic light scattering (Figure 1b). For identifying the statistical relations and associations between NMP sizes and observed biological effects, the Pearson correlation coefficients for all expression levels and other analyses (e.g., metabolic activity, ROS, and thiol content) were determined for single and repeated NMP treatments (Table 1). The cellular NMP uptake indicated a higher relative accumulation of smaller over larger (6.0 µm-sized) particles (Figure 1c). To visualize the NMP uptake into lung cells, cytoplasmic and intracellular membrane structures were stained, showing cellular localization of NMP in the cytoplasm around the cellular nucleus as shown in representative fluorescence and brightfield images (Figure 1d). NMP uptake was comparable between particle types and effective independent from lung cell population type and culture time as seen in representative images after several passages (Figure A1a). In addition, we quantified light scattering in NMP-treated cells using flow cytometry. Particle uptake was associated with increased intracellular vesicles, leading to higher light reflection. Particularly, the 2.0 µm-sized particle significantly increased side scatter signals (Figure 2a), validating the uptake quantification using fluorescence microscopy. Next, we measured alterations in cellular metabolism. We observed a slight increase in metabolic activity following NMP exposure, which was significant for the small particle between 0.2 and 1.0 µm. For repeated exposure, a significant decline was determined (Figure 2b). Representative images using the ROS indicator DCF showed a significantly higher intracellular ROS generation after exposure to plastic particles in contrast to untreated and H_2_O_2_-treated lung cells (Figure A1b). In contrast to the substantial ROS generation after NMP treatment, the thiol content was significantly decreased (Figure 2c). The increased ROS could be responsible for increased thiol consumption. We next investigated the effect of these findings on cell viability on a single-cell level using flow cytometry. Only moderate alterations of cell viability were found in cells 24 h after NMP exposure independent of particle size (Figure 2c), suggesting the increased ROS production and reduced thiol content (Figure 2d) to be of no cytotoxic consequences. However, NMP exposure altered the expression of the tumor suppressor protein p53, Bcl-2-associated X protein (*BAX*, a cofactor of p53), and the cell-cycle-related gene p21. p53 and BAX expression tended to increase after single and repeated NMP exposure for all particle sizes. In contrast, the observed lack of alterations of p21 after single incubation has reversed with increasing duration of NMP exposure except for 2.0 µm particles. Hypoxia-inducible factor 1α (*HIF1A*) expression was increased after single treatment for nano-sized particles but nearly unchanged for micro-sized particles and after repeated NMP exposure (Figure 2e), indicating an adaption of lung cells to NMP-induced effects.

### 3.2. NMP-Affected Stress Signaling in Primary Lung Epithelial Cells

**Figure 3:**
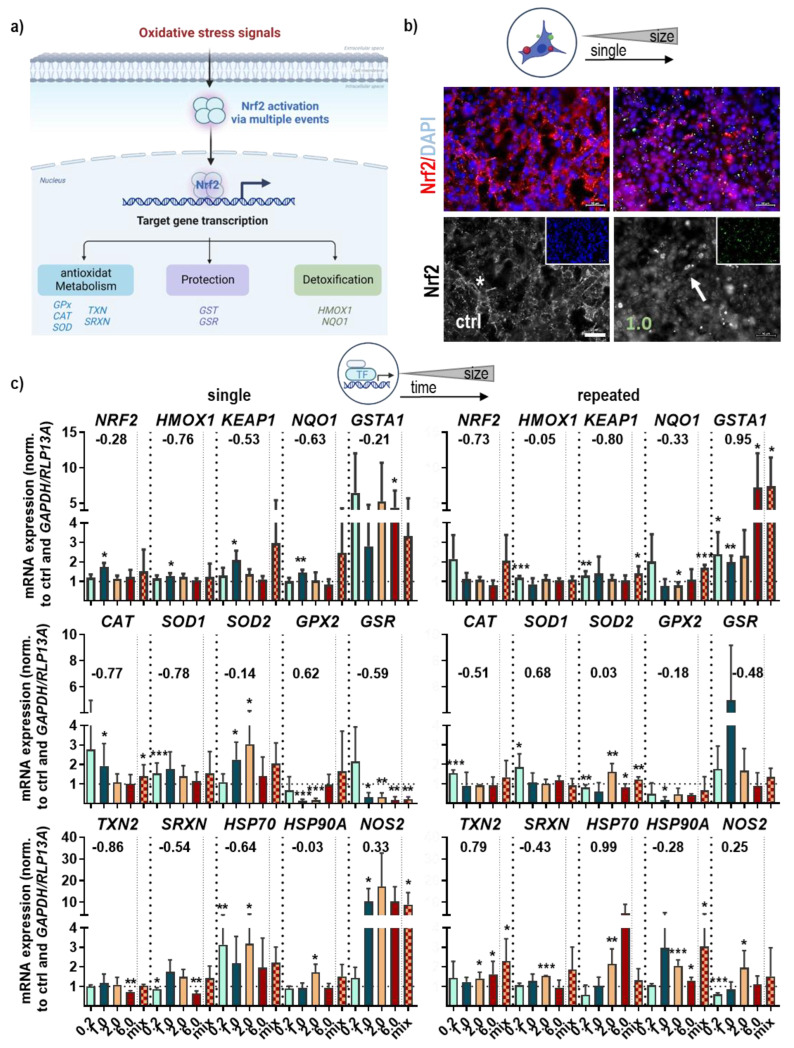

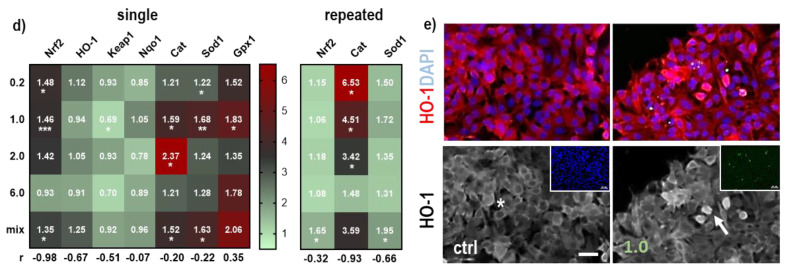
**Activation of oxidative stress signaling in murine lung epithelial cells after NMP exposure.** (**a**) Scheme of Nrf2 pathway activation and target gene expression; (**b**) representative images of cellular Nrf2 localization in untreated cells (ctrl; star, cytoplasm), and cells exposed to 1.0 µm-sized NMP (arrow, nuclear staining); (**c**) expression values of *Nrf2, HMOX1, Keap1 NQO1,* and *GSTA1* mRNA after single (left) and repeated (right) treatment using qPCR (upper panel), expression values of *CAT, SOD1, SOD2, GPx2, GSR* (middle panel), and *TXN2, SRXN, HSP70, HSP90A, iNOS* (bottom panel) were measured using qPCR after single and repeated NMP exposure; (**d**) heatmap of Nrf2 signaling and downstream targets by protein expression quantification; (**e**) representative images of cellular HO-1 localization in untreated cells (ctrl; star, cytoplasm) and cells exposed to 1.0 µm-sized NMP (arrow, nuclear staining) with nuclei counterstained with DAPI (blue). Data were normalized to *GAPDH/RLP13A* and untreated controls (ctrl) and presented as mean + SE. Statistical analysis was done by unpaired, two-tailed *Student*’s *t*-test with * *p* < 0.05, ** *p* < 0.01, and *** *p* < 0.001. Scale bars are 50 µm (**b**,**e**).

The literature shows that the cellular uptake of nanoparticles generates oxidative stress in several types of cell lines. Until now, several studies have identified changes in the nuclear factor-E2-related transcription factor 2 (Nrf2)-Keap1 signaling (Figure 3a) in models of respiratory diseases [26]. To identify the role of this pathway in our study, Nrf2 protein distribution and quantification, along with gene transcript levels and its downstream signaling, were analyzed following NMP treatment using immunofluorescence microscopy, qPCR, and WES (capillary-based protein gel electrophoreses). We observed a strong nuclear translocation of Nrf2 following exposure to 1.0 µm NMP (arrow in Figure 3b). This result was found independent of NMP size (data not shown), supporting the fact of an Nrf2 signaling activation following NMP exposure. Nrf2 plays an important role in regulating ARE-mediated expression of phase II detoxifying antioxidant genes. Therefore, we quantified the expression levels of *Nrf2*, heme oxygenase 1 (*HMOX1*), and NAD(P)H quinone oxidoreductase 1 (*NQO1*), two antioxidant proteins, as well as Keap1, a redox-regulated substrate adapter protein, which were modestly regulated in an NMP size-dependent fashion after single and repeated treatment (Figure 3c). At the same time, the glutathione S-transferase A1 (*GSTA1)* expression level was significantly increased, suggesting the protection of cells from ROS and peroxidation products in response to NMP treatment. Due to the antioxidant activity of several downstream targets of Nrf2 to protect cells from ROS-induced oxidative stress, we quantified the expression levels of catalase (*CAT*) and superoxide dismutase 1 and 2 (*SOD1/2*), which tended to be upregulated in an NMP size-dependent fashion. By contrast, glutathione peroxidase 2 (*GPx2*) was significantly down-regulated after single and repeated NMP exposure. In turn, glutathione reductase (*GSR*) was down-regulated upon single (1 week) NMP treatment but profoundly upregulated when cells were exposed over 4 weeks to NMP particles. Moreover, qPCR analysis of several targets such as thioredoxin 2 (*TXN2*), sulfiredoxin (*SRXN*), heat shock proteins (*HSP*) 70A and 90, and nitric oxide synthase 2 (inducible, *iNOS*) was divergently regulated upon NMP exposure, indicating a regulation of these targets through a redox-sensitive mechanism. The protein expression levels of Nrf2 were altered similarly to Nrf2 transcript levels. Other downstream targets were more modestly changed, except for Cat, Sod1, and GPx1, which were mostly upregulated after single or repeated NMP exposure (Figure 3d). Nuclear translocation of HO-1 protein following NMP treatment was shown in representative images using fluorescence microscopy (arrows in Figure 3e).

### 3.3. Structural and Functional Signaling Changes in NMP-Treated Lung Cells

**Figure 4:**
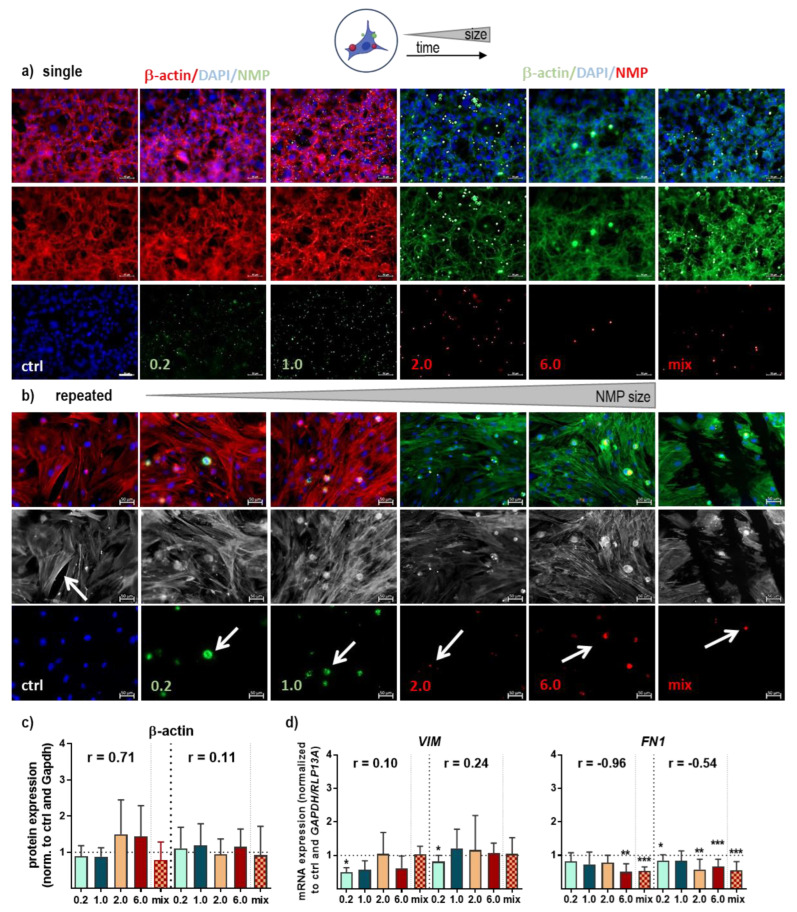
**Alteration of cell structure following NMP exposure.** (**a**,**b**) Study scheme and representative images showing cells that were grown on glass coverslips, incubated with selected NMP, fixed, and subjected to fluorescent labeling of actin stress fibers using FITC (green) or FlashRed-phalloidin (red) after single (**a**) and repeated NMP exposure ((**b**), arrows showed transient breakdown of the cytoskeleton in cells with NMP) with or without nuclear counterstaining (DAPI, blue); (**c**) protein expression level of β-actin measured by WES; (**d**) gene expression levels of *VIM*, and *FN1* quantified by qPCR. Data were normalized to *GAPDH/RLP13A* and untreated controls (ctrl) and presented as mean + SE. Statistical analysis was done by unpaired, two-tailed *Student*’s *t*-test with * *p* < 0.05, ** *p* < 0.01, and *** *p* < 0.001. Scale bars are 50 µm.

**Figure A2:**
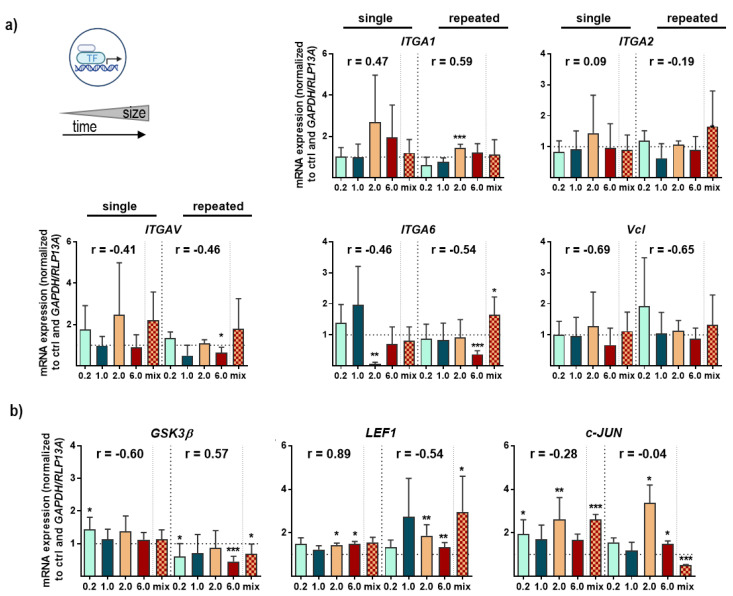
(**a**,**b**) Study scheme (top) and expression values of *ITGA1, ITGA2, ITGAV, ITGA6,* and *VCL* (**a**) and *GSK3β, LEF1,* and *c-JUN* (**b**) were quantified using qPCR after single and repeated NMP exposure. Data were normalized to *GAPDH/RLP13A* and untreated controls (ctrl) and presented as mean + SE. Statistical analysis was done by unpaired, two-tailed *Student*’s *t*-test with * *p* < 0.05, ** *p* < 0.01, and *** *p* < 0.001.

One role of the dynamic actin cytoskeleton is the spatial and temporal regulation of the β-catenin complex between adherent cell clusters. Consequently, we investigated structural target expression and changes following NMP exposure using qPCR, immunofluorescence microscopy, and protein analysis. While the β-actin expression level of *ACTB* mRNA (data not shown), as well as the staining of the cytoskeleton, was unchanged after single NMP exposure (Figure 4a), we found apparent effects following repeated NMP exposure. The cytoskeletal actin filaments were transiently disrupted along with a loss of stress fibers in cells that had accumulated plastic particles (arrows, Figure 4b). Using protein expression analyses (WES), however, we found no differences in absolute expression levels of β-actin (Figure 4c). Next, we quantified the expression level of the intermediate filament protein vimentin (VIM), and the multidomain protein fibronectin (FN) 1. mRNA expression levels of *VIM* were slightly down-regulated for 0.2 µm, 1.0 µm, and 6.0 µm NMP. Following single and repetitive treatment, all other sizes yielded comparable results to control conditions, except for 0.2 µm. In addition, we observed a prolonged down-regulation of the pericellular fibrinogen matrix as shown by *FN1* mRNA quantification (Figure 4d). The NMP effects on adhesive integrin complex and associated proteins, including integrins and vinculin, were quantified using qPCR showing differentially expression levels between experimental groups compared to untreated control (Figure A2a).

**Figure 5:**
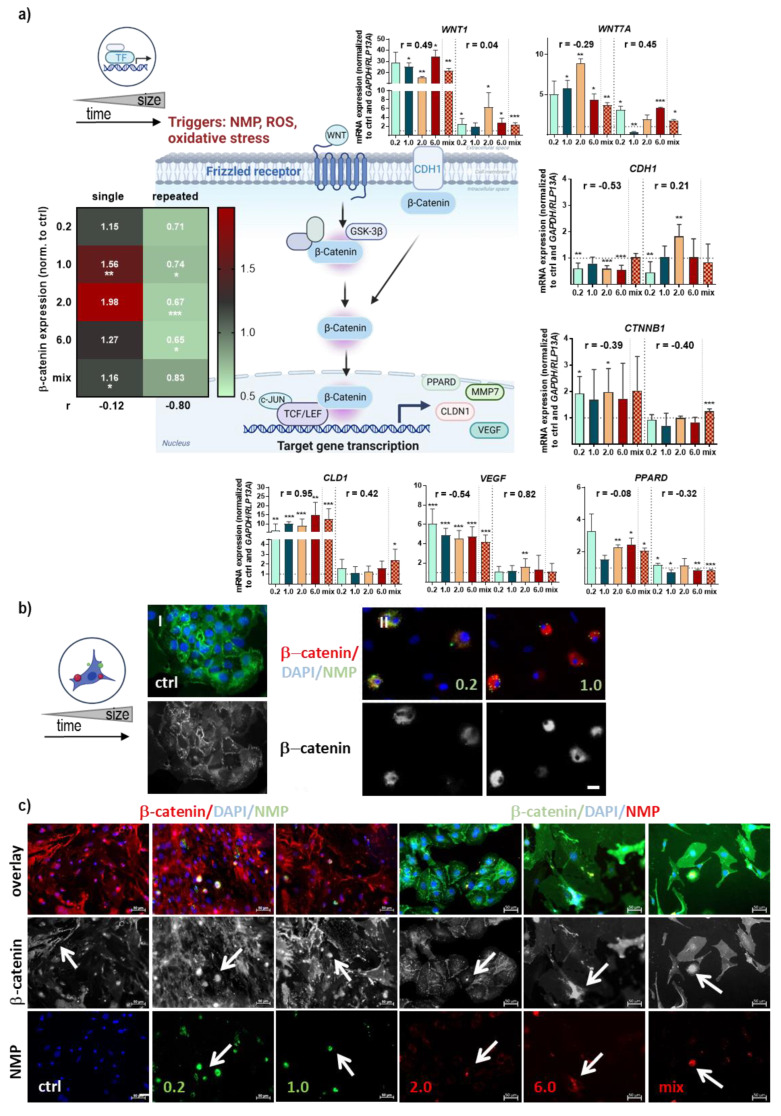
**Effects on β-catenin pathway following NMP exposure in lung cells.** (**a**) Study scheme and schematic representation of β-catenin signaling along with gene expression levels of *WNT1/7A, CDH1, CTNNB1, CLD1, VEGF*, and *PPARD* as measured by qPCR, and heatmap of β-catenin protein level quantified by WES; (**b**) representative images of distribution characteristics of β-catenin (green) in untreated lung cells (ctrl) with predominant cell border and cytoplasmic staining as compared to predominant cytoplasmic staining after single NMP exposures to 0.2 µm and 1.0 µm particles; (**c**) representative images of distribution characteristics of β-catenin (red) in untreated (ctrl) and NMP exposed lung cells (red or green particle fluorescence) with nuclei counterstained using DAPI (blue) and β-catenin being marked with arrows. Data were normalized to *GAPDH/RLP13A* and untreated controls (ctrl) and presented as mean + SE. Statistical analysis was done by unpaired, two-tailed *Student*’s *t*-test with * *p* < 0.05, ** *p* < 0.01, and *** *p* < 0.001. Scale bars are 50 µm (ctrl in **b**,**c**) and 20 µm (0.2/1.0 in **b**).

The dual-function protein β-catenin is a structural cell adhesion protein that binds intracellularly to cadherins and plays a signaling role as a downstream mediator of the Wnt signaling pathway (scheme Figure 5a). Through qPCR and WES analyses, we showed a substantial increase in β-catenin (*CTNNB1*) gene and protein level after single NMP treatment, in contrast to repeated exposure. This was accompanied by a slight decrease in E-cadherin (*CDH1*) expression after single treatment in contrast to repeated exposure, along with a strong increase of *WNT1* and *WNT7a* expression. Phosphorylated glycogen synthase kinase (GSK) 3β promotes the phosphorylation of β-catenin at key Ser/Thr residues by modulating its degradation and nuclear translocation [31]. The analyses revealed that the mRNA level of *GSK3β* was comparable between NMP-treated cells and controls, with a slight increase for 0.2 µm-sized NMP exposure. Consistent with previous data, a significant decrease in *GSK3β* mRNA was identified in repetitive NMP-treated lung cells. After nuclear β-catenin accumulation, it acts as a coactivator for transcription factors of the T-cell/lymphoid enhancer-biding factor (TCF/LEF) family. *LEF1* and *c-JUN,* as further activators downstream of β-catenin stabilization, showed similar results in single- and repeated-treated lung cells with slightly increased mRNA levels in the NMP-treated lung cells (Figure A2b). The mRNA levels of the tight junction protein claudin 1 (*CLDN1*) and vascular endothelial factor (*VEGF*) were significantly elevated in NMP-treated lung cells, particularly after single treatment and independent of particle size. For other molecules, such as the lipid-sensitive nuclear peroxisome proliferator-activated receptor δ (*PPARD*), mRNA expression increased after single treatment and decreased after prolonged NMP exposure, except for 0.2 µm NMP (Figure 5a). In addition, we observed an exposure-time-dependent increase in accumulation and nuclear staining of these targets using immunofluorescence, demonstrating an activation of the β-catenin pathway. The subcellular location of β-catenin showed stronger cytoplasmic expression following exposure to 0.2–1.0 µm NMP-treated cells (Figure 5b). Stronger membranous staining at cell–cell contacts was mainly observed in lung cells where NMP were not incorporated (ctrl) in contrast to a cytoplasmic and nuclear expression following long-term exposure to NMP-treated lung cells (arrows, Figure 5c).

### 3.4. Cytokine Secretion Profile Changes

**Figure 6:**
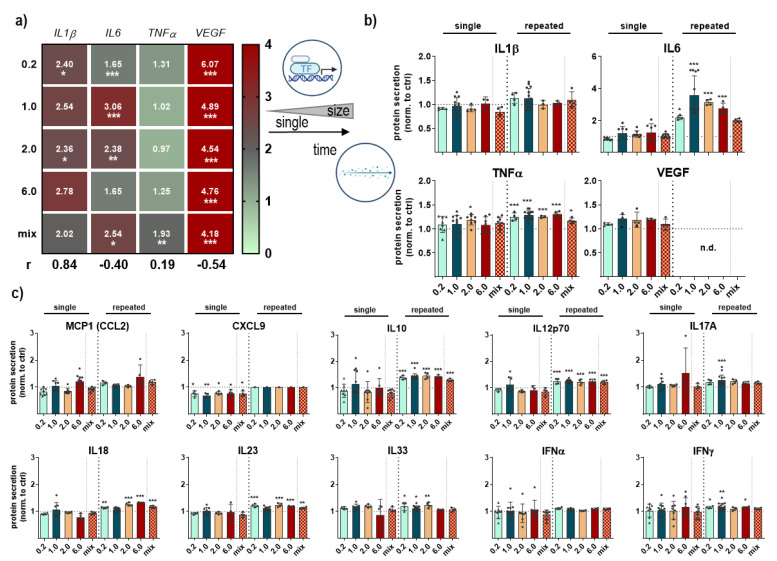
**Secretion profiles in NMP-treated lung cells**. (**a**) Expression levels of *IL1β*, *IL6*, *TNFα*, and *VEGF* measured using qPCR and shown in a heatmap after single NMP exposure; (**b**,**c**) multiplex protein release quantification after single and repeated NMP exposure in lung cell supernatants as shown in diagrams. Data were normalized to untreated controls (ctrl) and presented as mean + SE. Statistical analysis was done by unpaired, two-tailed *Student*’s *t*-test (*n* > 6) with * *p* < 0.05, ** *p* < 0.01, and *** *p* < 0.001.

The last aim of the study was to identify the chemokine, cytokine, and growth factor secretion pattern after NMP exposure in primary lung cells. First, we quantified the induction of pro-inflammatory cytokine expression using qPCR in primary lung cell subpopulations. NMP treatment induced a significant upregulation of interleukin 1β (*IL1β*), *IL6,* and *VEGF* after single NMP exposure in all experimental groups. The expression of the tumor necrosis factor *TNFα*, another mediator of an inflammatory response, has shown a slight decrease except for the NMP mix (Figure 6a). We identified the presence of cytokines which seem to be partly regulated at the translational level by qPCR. mRNA often is also regulated on a post-transcriptional level. To complement these data, we further analyzed the secretion of inflammation-related chemokines and cytokines using multiplex bead-based flow cytometry. In particular, IL6 and TNFα secretion into cell culture supernatants was significantly increased in NMP-treated lung cells (Figure 6b). At the same time, the strong regulation found for IL1β and VEGF after single NMP treatment seen with the qPCR results was not confirmed in cellular secretion analysis. In general, the repeated NMP exposure had a more pronounced effect on the secretion profile than the single treatment, as shown in single diagrams for all cytokines investigated here (Figure 6c).

## 4. Discussion

Preliminary data has demonstrated the presence of polymeric nano- and microplastic particles (NMP) in humans [17], particularly in the gastrointestinal tract [32], liver [33], placenta [34], and lung tissue [35]. Airborne pollutants are an important source of human exposure and are often inhaled through the lungs. The maternal pulmonary exposure to polystyrene (PS) nanoparticles was demonstrated, resulting in the translocation of plastic particles to placental and fetal tissues in rats [36]. Due to an increased awareness of microfibers and artificial microplastic in the air and possible consequences for human health, which have already been seen in the case of ultrafine particles [37], studies have aimed to identify and address toxic implications. Thus, the major aim of our study was to investigate several biological consequences following single and repeated NMP exposure in airway cells. Most previous studies investigating NMP effects on airway cells used cell lines such as alveolar A549 [5,38], bronchial BEAS [39,40,41], or lung cancer Calu-3 [42] cells, which are deficient in functional characteristics of normal lung cells [8]. Therefore, we used primary lung epithelial cells, freshly isolated from mice, to study biological responses in lung cells [43].

The composition, size, and shape of NMP significantly influence their retention in the lungs and targeting properties. Plastic particles with fragmented or fiber shapes are more abundant in environmental exposure scenarios than spherical particles of controlled size [44]. However, spherical particles are an advantageous proof-of-concept model for detecting hazardous NMP effects in biological targets. Most nanoparticles for biomedical applications, such as cancer treatment, exhibit a diameter of ~100 nm [35]. Additionally, NMP size impacts the rate and extent of uptake from the lungs, which was found for particle sizes ranging from 50 to 900 nm [45]. Microparticles with a mean volume diameter of about 3 μm and mass median aerodynamic diameter of around 6 μm were found to be the best suitable for pulmonary delivery as a therapeutic agent in diseases [46]. It is generally believed that aerosol particles between 5 and 10 μm are preferentially deposited into the oropharynx and large conducting airways. In contrast, particles between 1 and 5 μm are deposited in the small airways and alveoli [47]. To assess the cellular internalization and uptake of NMP, we used commercially available spheric polystyrene NMP of different sizes ranging from nano- (200 nm) to micro-sized particles (1–6 µm). Successful incorporation of NMP, all containing a fluorescent dye, into lung cells was shown by high-content imaging analyses. In vitro, a continuous penetration and intracellularly accumulation of small insoluble nano- and micro-sized theranostic particles were shown to treat several diseases [48], and in pulmonary tissue overcoming the epithelial barrier [7,49]. The presence of polymeric particles was reported, especially for PS particles, ranging from 4 to 30 µm in human liver tissue samples of individuals with liver cirrhosis. Surprisingly, some NMP particles identified had altered surfaces suggesting that particles had been deposited in the organ for a long time and exposed to possible biochemical processes [33].

Due to the lack of analytical techniques, little information regarding airborne NMP is available. Previously, an indoor air concentration of 3–15 particles/m^3^ [7] and 0.4–60 particles/m^3^ [46] was found. The use of a realistic concentration is crucial [50] and was indicated in the range of 0.5–0.86 µg/cm^2^ for inhalation treatment experiments of pulmonary cancer [47]. Studies have been performed with different NMP levels on cerebral and epithelial human cells (around 10 µg/mL) [51]; on human epithelial, microvascular endothelial (HMEC), and hepatoma cells, and macrophages (6–100 µg/mL) [41]; in hepatocarcinoma liver [52], and intestinal cells (1–200 µg/mL) [53]; in zebrafish embryos [54]; and in human lung epithelial cells such as A549 (100 µg/mL) [5,55]. Nevertheless, PS particles did not cause toxicity and decreased cellular viability at 20 and 50 µg/mL but at 200 µg/mL in THP1 monocytes [56] or at 120 mg/mL in endothelial cells [57]. To facilitate studies on biological effects using different particle sizes, we applied NMP concentrations at 100 mg/mL.

According to previous studies, exposures lasting for at least four weeks can be considered long-term (chronic) exposure [58,59,60], which is particularly more realistic, as present with our environment. Concomitant with previous findings [61], a significant decrease in metabolic activity was found only after repetitive NMP exposure but not after single exposure. On the cellular level, apoptosis and mitochondrial dysfunction are consequences of NMP exposure as found in vitro in cell cultures and in vivo in animals [62]. Previous data suggested that prolonged exposure to nano-sized particles leads to cytotoxicity at low doses, and a particle-induced induction of cell death may be involved in the observed pro-inflammatory action of NMP. Cell viability results showed that none of the experimental NMP groups studied here did lead to a strong reduction of cell viability or cytolysis. A decrease in cell number and an increase in interleukins were significant in monocytes, as shown with PS and silica nanoparticles in short-term and long-term exposures at 50 µg/mL [56], which was also evident in our cell model. Our data are consistent with previous studies, where an increase in the secretion of tumor necrosis factor (TNF) α following treatment with < 1 µm-sized PS at a concentration of 500 µg/mL was observed, together with a change in the interleukin (IL) 6 secretion following < 10 µm-sized PS particle exposure [63,64]. Detection of the spectrum of pro- and anti-inflammatory cytokines has become important in the study of several diseases in humans. An elevated inflammation level by the prolonged release of certain pro-inflammatory cytokines (e.g., IL1β, IL6, and TNFα) leads to severe problems [65]. It can create favorable environments for non-healing wounds [66] or tumor progression [67]. Moreover, it was demonstrated that nano-sized particles are taken up preferentially by lung cells stimulated cytokine and chemokine production [68]. Additionally, the pro-inflammatory response may occur due to permanent exposure to and accumulation of NMP.

The penetration of the cell membrane in response to cellular NMP uptake is strongly linked to biological responses on the single-cell level, such as cytotoxicity, apoptosis, cellular damage, and activation of signal transduction [69]. Several pathophysiological mechanisms have been described to clarify the toxicity of airborne NMP. Previous data confirmed that oxidative stress is one of the mechanisms of toxic effects at the cellular level [51]. An increase in ROS production following exposure to PS nanoparticles resulted in DNA damage and the increased formation of micronuclei and nuclear buds in a human fibroblast cell line [70]. Additionally, an elevated ROS level was detected after NMP exposure in human epithelial cells [71] and in mice exposed to NMP mixtures, while pre-treatment with antioxidants reversed the effects [72]. In contrast, minor changes in different genotoxicity-related biomarkers were observed in the human intestinal Caco-2 cells after long-term exposure to PS-NMP, indicating a lack of DNA damage or oxidative stress [53]. Consequently, we measured the ROS levels and verified an increase in intracellular ROS generation after NMP uptake in lung cells. In pathological conditions, the harmful effects of ROS can overwhelm the antioxidant system. Our findings of activation of the key regulatory transcription factor Nrf2 pathway showed a positive correlation with intracellular ROS level and oxidative stress response. We found elevated levels of the thiol metabolism-associated detoxifying enzyme glutathione S-transferase, hinting at an increase in the cellular detoxification caused by the intrinsic formation of ROS and products of oxidative stress by conjugating with glutathione. A potent antioxidant, neutralizing ROS, are protein thiols that are characterized by a sulfhydryl (SH-) group [73]. Reduced glutathione (GSH) is a major member that maintains the redox balance [74,75]. In contrast to increased thiol levels in human cell lines in response to NMP exposure [30], we observed decreased levels of thiols in lung cells suggesting less strong effects on viability as shown in Figure 2b,d. The cell viability was only decreased after repeated NMP exposure, promoting intracellular ROS production at low cytotoxicity. To examine the defense mechanism, downstream targets of Nrf2 signaling such as heme oxygenase 1, superoxide dismutase (SOD) 1 and 2, and catalase were increased, suggesting that NMP-generated oxidative stress could be considered as a trigger of oxidative stress-dependent signaling pathways mediated by Nrf2 [76]. An upregulation of SOD1 has been documented in microplastic-treated copepod *P. nana* [76]. A recent long-term study of NMP exposure found significant changes in the expression levels of HMOX1 and SOD2 transcripts in human intestinal cells [53]. Generally, the Nrf2 pathway activates cellular rescue pathways against oxidative injury, inflammation, immunity, apoptosis, and carcinogenesis [77]. Intriguingly, the nuclear translocation of HO-1 found in our study was also linked to tumorigenesis and tumor progression in lung, prostate, head, and neck squamous cell carcinomas and chronic myeloid leukemia [78]. Additionally, another study indicates the potential role of oxidative stress in the mechanism of nanoplastics-induced lung injuries with several key genes, such as transcription factors [79]. The reports and our findings suggested that repeated exposure to non-cytotoxic NMP concentrations increases the stress-related responses of the exposed cells. Therefore it could induce stress-related effects such as pulmonary fibrosis or carcinogenesis [49]. It was further found that PS-NMP leads to cardiovascular toxicity in rats by inducing cardiac fibrosis via activating the Wnt/β-catenin pathway and myocardium apoptosis triggered by oxidative stress [80]. The dual-function protein β-catenin is an important effector in the canonical wingless (Wnt)/β-catenin signaling where Wnt ligands bind to a frizzled Wnt receptor [81]. In a meta-analysis of 22 studies with ca. 2300 case reports, cytosolic β-catenin stabilization with nuclear translocation and accumulation was strongly associated with poor prognosis in hepatocellular carcinoma [82], colorectal cancer [83], and non-small cell lung cancer [84]. In this regard, we also found significant alterations in β-catenin localization, actin distribution, and fibrinogen expression indicative of modulated cytoskeleton and cellular structures.

## 5. Conclusions

In conclusion, due to our findings, NMP accumulation in murine lung cells supports intracellular ROS formation along with modest alterations in cytotoxicity and apoptosis. In contrast, NMP exposure changed the transcriptional signature of the Nrf2-driven oxidative stress response and β-catenin signaling along with modulation of inflammatory mediators (e.g., cytokines) and cytoskeletal factors (e.g., β-actin), particularly following repeated NMP deposition. To obtain more data on toxic NMP effects, further in vivo studies using environmental plastic mixtures must be conducted.

## Figures and Tables

**Table 1:** Pearson (r) correlation coefficients for several types of analyses for single and repeated NMP effects with the goal of identifying statistical relations between plastic particle sizes and biological effects observed, sorted for descending r-values, showing gene expression after single exposure (top left), gene expression after repeated exposure (top right), protein expression after single (bottom left) repeated (middle bottom right) exposure, and cellular processes (bottom right) such as uptake, metabolic activity, ROS, and thiol content.

Single (Gene Expression)				Repeated (Gene Expression)			
Target	PCC (r)	Significance		Target		PCC (r)	Significance
*FN1*	−0.96	*		*KEPA1*		−0.80	ns
*TXN2*	−0.86	ns		*NRF2*		−0.73	ns
*P53*	−0.82	ns		*VCL*		−0.65	ns
*SOD1*	−0.78	ns		*FN1*		−0.54	ns
*CAT*	−0.77	ns		*ITGA6*		−0.54	ns
*HMOX1*	−0.76	ns		*LEF1*		−0.54	ns
*VCL*	−0.69	ns		*CAT*		−0.51	ns
*HSP70*	−0.64	ns		*GSR*		−0.48	ns
*NQO1*	−0.63	ns		*HIF1A*		−0.47	ns
*GSK3B*	−0.60	ns		*ITGA5*		−0.46	ns
*GSR*	−0.59	ns		*SXRN*		−0.43	ns
*SRXN*	−0.54	ns		*CTNNB1*		−0.40	ns
*VEGF*	−0.54	ns		*NQO1*		−0.33	ns
*CDH1*	−0.53	ns		*PPARD*		−0.32	ns
*KEAP1*	−0.53	ns		*HSP90A*		−0.28	ns
*HIF1A*	−0.50	ns		*ITGA2*		−0.19	ns
*ITGA6*	−0.46	ns		*GPx2*		−0.18	ns
*ITGAV*	−0.41	ns		*HMOX1*		−0.05	ns
*IL6*	−0.41	ns		*C-JUN*		−0.04	ns
*CTNNB1*	−0.39	ns		*SOD2*		0.03	ns
*WNT7A*	−0.29	ns		*WNT1*		0.04	ns
*C-JUN*	−0.28	ns		*P53*		0.18	ns
*NRF2*	−0.28	ns		*CDH1*		0.21	ns
*GSTA1*	−0.21	ns		*VIM*		0.24	ns
*P21*	−0.16	ns		*NOS2 (INOS)*		0.25	ns
*SOD2*	−0.14	ns		*CLD1*		0.42	ns
*PPARD*	−0.08	ns		*BAX*		0.44	ns
*HSP90A*	−0.03	ns		*WNT7A*		0.45	ns
*BAX*	0.06	ns		*GSK3B*		0.57	ns
*ITGA2*	0.09	ns		*P21*		0.57	ns
*VIM*	0.10	ns		*ITGA1*		0.59	ns
*TNFα*	0.19	ns		*GSTA1*		0.59	ns
*NOS2 (INOS)*	0.33	ns		*SOD1*		0.68	ns
*ITGA1*	0.47	ns		*TXN2*		0.79	ns
*WNT1*	0.49	ns		*HSP70A*		0.99	**
*GPX2*	0.62	ns					
*IL1β*	0.84	ns				r > 0.9 or r < −0.9	strong
*LEF1*	0.89	ns				r > 0.7 or r < −0.7	moderate
*CLD1*	0.95	**				0.7 > r < −0.7	weak
**Single (Protein Expression)**				**Repeated (Protein Expression)**			
**Target**	**PCC (r)**	***p*** **-Value**	**Significance**	**Target**	**PCC (r)**	***p*** **-Value**	**Significance**
Nrf2	−0.98	0.02	*	Cat	−0.93	0.05	*
HO-1	−0.67	0.33	ns	β-catenin	−0.80	0.2	ns
Cldn1	−0.54	0.46	ns	Sod1	−0.66	0.34	ns
Keap1	−0.51	0.49	ns	Nrf2	−0.32	0.67	ns
Sod1	−0.22	0.78	ns	β-actin	0.11	0.89	ns
integrin	−0.21	0.79	ns				
Cat	−0.20	0.80	ns				
β-catenin	−0.12	0.23	ns				
Nqo1	−0.07	0.93	ns				
Vcl	0.32	0.68	ns				
Gpx1	0.35	0.65	ns				
Wnt	0.59	0.41	ns				
β-actin	0.71	0.29	ns				
**Cellular Process**							
**Target**		**PCC (r)**		***p*** **-Value**		**Significance**	
NMP uptake		−0.53		0.8		ns	
Metabolic activity (s)		−0.34		0.66		ns	
Metabolic activity (r)		−0.24		0.76		ns	
ROS		−0.17		0.83		ns	
Thiol content		−0.39		0.61		ns	

## Data Availability

The datasets supporting the conclusions of this article are included within the article, and can be retrieved from the corresponding author upon reasonable request.

## Appendix A

**Table A1:** Murine gene-specific primers used in quantitative real-time PCR.

Gene Name	Alias with Gene ID	Primer Sequences (3′-5′)
nuclear factor erythroid 2-related factor 2	*NRF2_*NM_010902	GAG TCG CTT GCC CTG GAT ATC TCA TGG CTG CCT CCA GAG AA
heme oxygenase 1	*HMOX1*_NM_010442	TGA AGC AGG CAT CTG AGG G CGA AGG TGG AAG AGT GGG AG
NAD(P)H dehydrogenase [quinone] 1	*NQO1*_NM_008706	GGC ATC CAG TCC TCC ATC AA GTT AGT CCC TCG GCC ATT GTT
kelch-like ECH-associated protein 1	*KEAP1*_NM_016679	CGG GGA CGC AGT GAT GTA TG TGT GTA GCT GAA GGT TCG GTT A
catalase	*CAT*_NM_009804	CAG AGA GCG GAT TCC TGA GAG A CTT TGC CTT GGA GTA TCT GGT GAT
superoxide dismutase [Cu-Zn]	*SOD1*_NM_011434	GAA ACA AGA TGA CTT GGG CAA AG TTA CTG CGC AAT CCC AAT CA
glutathione peroxidase 1	*GPX2*_NM_030677	GTG GCG TCA CTC TGA GGA ACA CAG TTC TCC TGA TGT CCG AAC TG
glutathione-disulfide reductase	*GSR*_NM_010344	TCG GAA TTC ATG CAC GAT CA GGC TCA CAT AGG CAT CCC TTT
tumor necrosis factor-alpha	*TNFA*_NM_013693	TCT CAT GCA CCA CCA TCA AGG ACT ACC ACT CTC CCT TTG CAG AAC TCA
interleukin 1β	*IL1β*	GCA ACT GTT CCT GAA CTC AAC T ATC TTT TGG GGT CCG TCA ACT
interleukin 6	*IL6*_NM_031168	ATC CAG TTG CCT TCT TGG GAC TGA TAA GCC TCC GAC TTG TGA AGT GGT
β actin	*ACTB*_NM_007393	TTG CTG ACA GGA TGC AGA AG ACA TCT GCT GGA AGG TGG AC
heat shock protein 70	*HSP70*_NM_010479	GGC TGA CAA GAA GAA GGT GC CTG GTA CAG CCC ACT GAT GA
heat shock protein 90α	*HSP90A*_NM_010480	GAC GCT CTG GAT AAA ATC CGT T TGG GAA TGA GAT TGA TGT GCA G
vinculin	*VCL*_NM_009502	GCT TCAGTC AGACCC ATA CTC G AGG TAA GCA GTA GGT CAG ATG T
focal adhesion kinase	*FAK/PTK2*_NM_007982	GAG TAC GTC CCT ATG GTG AAG G CTC GAT CTC TCG ATG AGT GCT
integrin A1	*ITGA1*_ NM_001033228	GAC AGC CCT TGG AAT AGA CAC GTT GTC ATG CGA TTC TCC ATC A
integrin A2	*ITGA2*_NM_008396	TGT CTG GCG TAT AAT GTT GGC TGC TGT ACT GAA TAC CCA AAC TG
integrin A5	*ITGA5*_NM_008402	TGC AGT GGT TCG GAG CAA C TTT TCT GTG CGC CAG CTA TAC
integrin A6	*ITGA6*_NM_008397	GGG ATC GTC CGT GTA GAA CAA TCT CTC CAC CAA CTT CAT AGG G
fibronectin 1	*FN1*_NM_010233	GCT CAG CAA ATC GTG CAG C CTA GGT AGG TCC GTT CCC ACT
vimentin	*VIM*_NM_011701	CGT CCA CAC GCA CCT ACA G GGG GGA TGA GGA ATA GAG GCT
wingless-type MMTV integration site 1	*WNT1*_NM_021279	CGACTGATCCGACAGAACCC CCATTTGCACTCTCGCACA
wingless-type MMTV integration site 7a	*WNT7A*_NM_009527	TCAGTTTCAGTTCCGAAATGGC CCCGACTCCCCACTTTGAG
β-catenin 1	*CTNNB1*_NM_007614	CCC AGT CCT TCA CGC AAG AG CAT CTA GCG TCT CAG GGA ACA
E-cadherin	*CDH1*_NM_009864	CAC CTG GAG AGA GGC CAT GT TGG GAA ACA TGA GCA GCT CT
lymphoid enhancer-binding factor 1	*LEF1*_NM_010703	GCC ACC GAT GAG ATG ATC CC TTG ATG TCG GCT AAG TCG CC
Jnk for c-jun N-terminal kinase α	*C-JUN*_AJ315350	GTCCTCCATAAATGCCTGTTCC GATGCAACCCACTGACCAGAT
peroxisome proliferator activator receptor delta	*PPARD*_NM_011145	TCCATCGTCAACAAAGACGGG ACTTGGGCTCAATGATGTCAC
claudin 1	*CLD1_*NM_016674	GGG GAC AAC ATC GTG ACC G AGG AGT CGA AGA CTT TGC ACT
vascular endothelial growth factor	*VEGF*_NM_001025250	AAC GAT GAA GCC CTG GAG TG GAC AAA CAA ATG CTT TCT CCG
glycogen synthase kinase 3 α	*GSK3B*_NM_019827	AAG CTC TGC GAT TTT GGC AGT GAG TTC TGG AGC ACG GTA GTA
glyceraldehyde 3-phosphate dehydrogenase	*GAPDH*_NM_001289726	CAT GGC CTC CAA GGA GTA AG TGT GAG GGA GAT GCT CAG TG
ribosomal protein 13A	*RPL13A*_NM_009438	AGC CTA CCA GAA AGT TTG CTT AC GCT TCT TCT TCC GAT AGT GCA TC

## References

[1] Blair R.M., Waldron S., Phoenix V., Gauchotte-Lindsay C. (2017). Micro- and Nanoplastic Pollution of Freshwater and Wastewater Treatment Systems. Springer Sci. Rev..

[2] Wagner S., Reemtsma T. (2019). Things We Know and Don’t Know About Nanoplastic in the Environment. Nat. Nanotechnol..

[3] Karakolis E.G., Nguyen B., You J.B., Rochman C.M., Sinton D. (2019). Fluorescent Dyes for Visualizing Microplastic Particles and Fibers in Laboratory-Based Studies. Environ. Sci. Technol. Lett..

[4] Allen S., Allen D., Phoenix V.R., Le Roux G., Durántez Jiménez P., Simonneau A., Binet S., Galop D. (2019). Atmospheric Transport and Deposition of Microplastics in a Remote Mountain Catchment. Nat. Geosci..

[5] Xu M., Halimu G., Zhang Q., Song Y., Fu X., Li Y., Li Y., Zhang H. (2019). Internalization and Toxicity: A Preliminary Study of Effects of Nanoplastic Particles on Human Lung Epithelial Cell. Sci. Total Environ..

[6] Prata J.C. (2018). Airborne Microplastics: Consequences to Human Health?. Environ. Pollut..

[7] Gasperi J., Wright S.L., Dris R., Collard F., Mandin C., Guerrouache M., Langlois V., Kelly F.J., Tassin B. (2018). Microplastics in Air: Are We Breathing It In?. Curr. Opin. Environ. Sci. Health.

[8] Manshoven S.S.A., Malarciuc C., Tenhunen A. (2022). Microplastic Pollution from Textile Consumption in Europe. Eionet Rep. ETC/CE.

[9] Saavedra J., Stoll S., Slaveykova V.I. (2019). Influence of Nanoplastic Surface Charge on Eco-Corona Formation, Aggregation and Toxicity to Freshwater Zooplankton. Environ. Pollut..

[10] Gopinath P.M., Saranya V., Vijayakumar S., Mythili Meera M., Ruprekha S., Kunal R., Pranay A., Thomas J., Mukherjee A., Chandrasekaran N. (2019). Assessment on Interactive Prospectives of Nanoplastics with Plasma Proteins and the Toxicological Impacts of Virgin, Coronated and Environmentally Released-Nanoplastics. Sci. Rep..

[11] Verla A.W., Enyoh C.E., Verla E.N., Nwarnorh K.O. (2019). Microplastic–Toxic Chemical Interaction: A Review Study on Quantified Levels, Mechanism and Implication. SN Appl. Sci..

[12] Hahladakis J.N., Velis C.A., Weber R., Iacovidou E., Purnell P. (2018). An Overview of Chemical Additives Present in Plastics: Migration, Release, Fate and Environmental Impact During Their Use, Disposal and Recycling. J. Hazard. Mater..

[13] Barbosa F., Adeyemi J.A., Bocato M.Z., Comas A., Campiglia A. (2020). A Critical Viewpoint on Current Issues, Limitations, and Future Research Needs on Micro-and Nanoplastic Studies: From the Detection to the Toxicological Assessment. Environ. Res..

[14] Chen S., Guo H., Cui M., Huang R., Su R., Qi W., He Z. (2020). Interaction of Particles with Mucosae and Cell Membranes. Colloids Surf. B Biointerfaces.

[15] Yang Y.F., Chen C.Y., Lu T.H., Liao C.M. (2019). Toxicity-Based Toxicokinetic/Toxicodynamic Assessment for Bioaccumulation of Polystyrene Microplastics in Mice. J. Hazard. Mater..

[16] Yong C.Q.Y., Valiyaveetill S., Tang B.L. (2020). Toxicity of Microplastics and Nanoplastics in Mammalian Systems. Int. J. Environ. Res. Public Health.

[17] Chang X., Xue Y., Li J., Zou L., Tang M. (2020). Potential Health Impact of Environmental Micro- and Nanoplastics Pollution. J. Appl. Toxicol..

[18] Elsaesser A., Howard C.V. (2012). Toxicology of Nanoparticles. Adv. Drug Deliv. Rev..

[19] Van Cauwenberghe L., Janssen C.R. (2014). Microplastics in Bivalves Cultured for Human Consumption. Environ. Pollut..

[20] Stock V., Laurisch C., Franke J., Donmez M.H., Voss L., Bohmert L., Braeuning A., Sieg H. (2021). Uptake and Cellular Effects of Pe, Pp, Pet and Pvc Microplastic Particles. Toxicol. In Vitro.

[21] Stock V., Bohmert L., Lisicki E., Block R., Cara-Carmona J., Pack L.K., Selb R., Lichtenstein D., Voss L., Henderson C.J. (2019). Uptake and Effects of Orally Ingested Polystyrene Microplastic Particles in Vitro and in Vivo. Arch. Toxicol..

[22] Salvi S. (2007). Health Effects of Ambient Air Pollution in Children. Paediatr. Respir. Rev..

[23] Stone A.L., Becker L.G., Huber A.M., Catalano R.F. (2012). Review of Risk and Protective Factors of Substance Use and Problem Use in Emerging Adulthood. Addict. Behav..

[24] Turner M.C., Krewski D., Diver W.R., Pope C.A., Burnett R.T., Jerrett M., Marshall J.D., Gapstur S.M. (2017). Ambient Air Pollution and Cancer Mortality in the Cancer Prevention Study Ii. Environ. Health Perspect..

[25] Amato-Lourenco L.F., Dos Santos Galvao L., de Weger L.A., Hiemstra P.S., Vijver M.G., Mauad T. (2020). An Emerging Class of Air Pollutants: Potential Effects of Microplastics to Respiratory Human Health?. Sci. Total Environ..

[26] Lee J., Jang J., Park S.M., Yang S.R. (2021). An Update on the Role of Nrf2 in Respiratory Disease: Molecular Mechanisms and Therapeutic Approaches. Int. J. Mol. Sci..

[27] Kim H.M., Lee D.K., Long N.P., Kwon S.W., Park J.H. (2019). Uptake of Nanopolystyrene Particles Induces Distinct Metabolic Profiles and Toxic Effects in Caenorhabditis Elegans. Environ. Pollut..

[28] Leslie H.A., van Velzen M.J.M., Brandsma S.H., Vethaak A.D., Garcia-Vallejo J.J., Lamoree M.H. (2022). Discovery and Quantification of Plastic Particle Pollution in Human Blood. Environ. Int..

[29] Leslie H.A., Depledge M.H. (2020). Where Is the Evidence That Human Exposure to Microplastics Is Safe?. Environ. Int..

[30] Da Silva Brito W.A., Singer D., Miebach L., Saadati F., Wende K., Schmidt A., Bekeschus S. (2022). Comprehensive in Vitro Polymer Type, Concentration, and Size Correlation Analysis to Microplastic Toxicity and Inflammation. Sci. Total Environ..

[31] Cheon S., Poon R., Yu C., Khoury M., Shenker R., Fish J., Alman B.A. (2005). Prolonged Beta-Catenin Stabilization and Tcf-Dependent Transcriptional Activation in Hyperplastic Cutaneous Wounds. Lab. Investig..

[32] Schwabl P., Koppel S., Konigshofer P., Bucsics T., Trauner M., Reiberger T., Liebmann B. (2019). Detection of Various Microplastics in Human Stool a Prospective Case Series. Ann. Intern. Med..

[33] Horvatits T., Tamminga M., Liu B., Sebode M., Carambia A., Fischer L., Puschel K., Huber S., Fischer E.K. (2022). Microplastics Detected in Cirrhotic Liver Tissue. EBioMedicine.

[34] Ragusa A., Svelato A., Santacroce C., Catalano P., Notarstefano V., Carnevali O., Papa F., Rongioletti M.C.A., Baiocco F., Draghi S. (2021). Plasticenta: First Evidence of Microplastics in Human Placenta. Environ. Int..

[35] Lee T.R., Choi M., Kopacz A.M., Yun S.H., Liu W.K., Decuzzi P. (2013). On the near-Wall Accumulation of Injectable Particles in the Microcirculation: Smaller Is Not Better. Sci. Rep..

[36] Fournier S.B., D’Errico J.N., Adler D.S., Kollontzi S., Goedken M.J., Fabris L., Yurkow E.J., Stapleton P.A. (2020). Nanopolystyrene Translocation and Fetal Deposition after Acute Lung Exposure During Late-Stage Pregnancy. Part. Fibre Toxicol..

[37] Moreno-Ríos A.L., Tejeda-Benítez L.P., Bustillo-Lecompte C.F. (2022). Sources, Characteristics, Toxicity, and Control of Ultrafine Particles: An Overview. Geosci. Front..

[38] Deville S., Penjweini R., Smisdom N., Notelaers K., Nelissen I., Hooyberghs J., Ameloot M. (2015). Intracellular Dynamics and Fate of Polystyrene Nanoparticles in A549 Lung Epithelial Cells Monitored by Image (Cross-) Correlation Spectroscopy and Single Particle Tracking. Biochim. Biophys. Acta.

[39] Lim S.L., Ng C.T., Zou L., Lu Y., Chen J., Bay B.H., Shen H.M., Ong C.N. (2019). Targeted Metabolomics Reveals Differential Biological Effects of Nanoplastics and Nanozno in Human Lung Cells. Nanotoxicology.

[40] Chiu H.W., Xia T., Lee Y.H., Chen C.W., Tsai J.C., Wang Y.J. (2015). Cationic Polystyrene Nanospheres Induce Autophagic Cell Death through the Induction of Endoplasmic Reticulum Stress. Nanoscale.

[41] Xia T., Kovochich M., Liong M., Zink J.I., Nel A.E. (2008). Cationic Polystyrene Nanosphere Toxicity Depends on Cell-Specific Endocytic and Mitochondrial Injury Pathways. ACS Nano.

[42] Paget V., Dekali S., Kortulewski T., Grall R., Gamez C., Blazy K., Aguerre-Chariol O., Chevillard S., Braun A., Rat P. (2015). Specific Uptake and Genotoxicity Induced by Polystyrene Nanobeads with Distinct Surface Chemistry on Human Lung Epithelial Cells and Macrophages. PLoS ONE.

[43] Gonzalez R.F., Dobbs L.G. (2013). Isolation and Culture of Alveolar Epithelial Type I and Type Ii Cells from Rat Lungs. Methods Mol. Biol..

[44] Garbuzenko O.M.G., Taratula O., Minko T. (2014). Inhalation Treatment of Lung Cancer: The Influence of Composition, Size and Shape of Nanocarriers on Their Lung Accumulation and Retention. Cancer Biol. Med..

[45] Mohammad A.K., Amayreh L.K., Mazzara J.M., Reineke J.J. (2013). Rapid Lymph Accumulation of Polystyrene Nanoparticles Following Pulmonary Administration. Pharm. Res..

[46] Alipour S., Montaseri H., Tafaghodi M. (2010). Preparation and Characterization of Biodegradable Paclitaxel Loaded Alginate Microparticles for Pulmonary Delivery. Colloids Surf. B Biointerfaces.

[47] Kuzmov A., Minko T. (2015). Nanotechnology Approaches for Inhalation Treatment of Lung Diseases. J. Control. Release.

[48] Patra J.K., Das G., Fraceto L.F., Campos E.V.R., Rodriguez-Torres M.D.P., Acosta-Torres L.S., Diaz-Torres L.A., Grillo R., Swamy M.K., Sharma S. (2018). Nano Based Drug Delivery Systems: Recent Developments and Future Prospects. J. Nanobiotechnol..

[49] Facciola A., Visalli G., Pruiti Ciarello M., Di Pietro A. (2021). Newly Emerging Airborne Pollutants: Current Knowledge of Health Impact of Micro and Nanoplastics. Int. J. Environ. Res. Public Health.

[50] Krug H.F. (2014). Nanosafety Research--Are We on the Right Track?. Angew Chem. Int. Ed. Engl..

[51] Schirinzi G.F., Perez-Pomeda I., Sanchis J., Rossini C., Farre M., Barcelo D. (2017). Cytotoxic Effects of Commonly Used Nanomaterials and Microplastics on Cerebral and Epithelial Human Cells. Environ. Res..

[52] Hesler M., Aengenheister L., Ellinger B., Drexel R., Straskraba S., Jost C., Wagner S., Meier F., von Briesen H., Buchel C. (2019). Multi-Endpoint Toxicological Assessment of Polystyrene Nano- and Microparticles in Different Biological Models in Vitro. Toxicol. In Vitro.

[53] Domenech J., de Britto M., Velázquez A., Pastor S., Hernández A., Marcos R., Cortés C. (2021). Long-Term Effects of Polystyrene Nanoplastics in Human Intestinal Caco-2 Cells. Biomolecules.

[54] Brun N.R., Koch B.E.V., Varela M., Peijnenburg W.J.G.M., Spaink H.P., Vijver M.G. (2018). Nanoparticles Induce Dermal and Intestinal Innate Immune System Responses in Zebrafish Embryos. Environ. Sci. Nano.

[55] Kang T., Park C., Lee B.J. (2016). Investigation of Biomimetic Shear Stress on Cellular Uptake and Mechanism of Polystyrene Nanoparticles in Various Cancer Cell Lines. Arch. Pharmacal Res..

[56] Mrakovcic M., Meindl C., Roblegg E., Frohlich E. (2014). Reaction of Monocytes to Polystyrene and Silica Nanoparticles in Short-Term and Long-Term Exposures. Toxicol. Res. (Camb).

[57] Mrakovcic M., Absenger M., Riedl R., Smole C., Roblegg E., Frohlich L.F., Frohlich E. (2013). Assessment of Long-Term Effects of Nanoparticles in a Microcarrier Cell Culture System. PLoS ONE.

[58] Domenech J., Marcos R. (2021). Pathways of Human Exposure to Microplastics, and Estimation of the Total Burden. Curr. Opin. Food Sci..

[59] Vales G., Rubio L., Marcos R. (2015). Long-Term Exposures to Low Doses of Titanium Dioxide Nanoparticles Induce Cell Transformation, but Not Genotoxic Damage in Beas-2b Cells. Nanotoxicology.

[60] Annangi B., Bach J., Vales G., Rubio L., Marcos R., Hernandez A. (2015). Long-Term Exposures to Low Doses of Cobalt Nanoparticles Induce Cell Transformation Enhanced by Oxidative Damage. Nanotoxicology.

[61] Schroter L., Ventura N. (2022). Nanoplastic Toxicity: Insights and Challenges from Experimental Model Systems. Small.

[62] Heddagaard F.E., Moller P. (2020). Hazard Assessment of Small-Size Plastic Particles: Is the Conceptual Framework of Particle Toxicology Useful?. Food Chem. Toxicol..

[63] Green T.R., Fisher J., Stone M., Wroblewski B.M., Ingham E. (1998). Polyethylene Particles of a ‘Critical Size’ Are Necessary for the Induction of Cytokines by Macrophages in Vitro. Biomaterials.

[64] Shanbhag A.S., Jacobs J.J., Black J., Galante J.O., Glant T.T. (1994). Macrophage/Particle Interactions: Effect of Size, Composition and Surface Area. J. Biomed. Mater. Res..

[65] Zhang J.M., An J. (2007). Cytokines, Inflammation, and Pain. Int. Anesthesiol. Clin..

[66] Wall I.B., Moseley R., Baird D.M., Kipling D., Giles P., Laffafian I., Price P.E., Thomas D.W., Stephens P. (2008). Fibroblast Dysfunction Is a Key Factor in the Non-Healing of Chronic Venous Leg Ulcers. J. Investig. Dermatol..

[67] Amsen D., Antov A., Flavell R.A. (2009). The Different Faces of Notch in T-Helper-Cell Differentiation. Nat. Rev. Immunol..

[68] Hardy C.L., Lemasurier J.S., Mohamud R., Yao J., Xiang S.D., Rolland J.M., O’Hehir R.E., Plebanski M. (2013). Differential Uptake of Nanoparticles and Microparticles by Pulmonary Apc Subsets Induces Discrete Immunological Imprints. J. Immunol..

[69] Claudia M., Kristin O., Jennifer O., Eva R., Eleonore F. (2017). Comparison of Fluorescence-Based Methods to Determine Nanoparticle Uptake by Phagocytes and Non-Phagocytic Cells in Vitro. Toxicology.

[70] Poma A., Vecchiotti G., Colafarina S., Zarivi O., Aloisi M., Arrizza L., Chichiricco G., Di Carlo P. (2019). In Vitro Genotoxicity of Polystyrene Nanoparticles on the Human Fibroblast Hs27 Cell Line. Nanomaterials.

[71] Hu M., Palic D. (2020). Micro- and Nano-Plastics Activation of Oxidative and Inflammatory Adverse Outcome Pathways. Redox Biol..

[72] Liang B., Zhong Y., Huang Y., Lin X., Liu J., Lin L., Hu M., Jiang J., Dai M., Wang B. (2021). Underestimated Health Risks: Polystyrene Micro- and Nanoplastics Jointly Induce Intestinal Barrier Dysfunction by Ros-Mediated Epithelial Cell Apoptosis. Part. Fibre Toxicol..

[73] Birben E., Sahiner U.M., Sackesen C., Erzurum S., Kalayci O. (2012). Oxidative Stress and Antioxidant Defense. World Allergy Organ. J..

[74] Aquilano K., Baldelli S., Ciriolo M.R. (2014). Glutathione: New Roles in Redox Signaling for an Old Antioxidant. Front. Pharmacol..

[75] He Y., Li J., Chen J., Miao X., Li G., He Q., Xu H., Li H., Wei Y. (2020). Cytotoxic Effects of Polystyrene Nanoplastics with Different Surface Functionalization on Human Hepg2 Cells. Sci. Total Environ..

[76] Jeong C.B., Kang H.M., Lee M.C., Kim D.H., Han J., Hwang D.S., Souissi S., Lee S.J., Shin K.H., Park H.G. (2017). Adverse Effects of Microplastics and Oxidative Stress-Induced Mapk/Nrf2 Pathway-Mediated Defense Mechanisms in the Marine Copepod Paracyclopina Nana. Sci. Rep..

[77] Cho H.Y., Reddy S.P., Kleeberger S.R. (2006). Nrf2 Defends the Lung from Oxidative Stress. Antioxid. Redox Signal..

[78] Yang Q., Wang W. (2022). The Nuclear Translocation of Heme Oxygenase-1 in Human Diseases. Front Cell Dev. Biol..

[79] Zhang T., Yang S., Ge Y., Wan X., Zhu Y., Li J., Yin L., Pu Y., Liang G. (2022). Polystyrene Nanoplastics Induce Lung Injury Via Activating Oxidative Stress: Molecular Insights from Bioinformatics Analysis. Nanomaterials.

[80] Li Z., Zhu S., Liu Q., Wei J., Jin Y., Wang X., Zhang L. (2020). Polystyrene Microplastics Cause Cardiac Fibrosis by Activating Wnt/Beta-Catenin Signaling Pathway and Promoting Cardiomyocyte Apoptosis in Rats. Environ. Pollut..

[81] Kawano Y., Kypta R. (2003). Secreted Antagonists of the Wnt Signalling Pathway. J. Cell Sci..

[82] Chen J., Liu J., Jin R., Shen J., Liang Y., Ma R., Lin H., Liang X., Yu H., Cai X. (2014). Cytoplasmic and/or Nuclear Expression of Beta-Catenin Correlate with Poor Prognosis and Unfavorable Clinicopathological Factors in Hepatocellular Carcinoma: A Meta-Analysis. PLoS ONE.

[83] Gao Z.H., Lu C., Wang M.X., Han Y., Guo L.J. (2014). Differential Beta-Catenin Expression Levels Are Associated with Morphological Features and Prognosis of Colorectal Cancer. Oncol. Lett..

[84] Li X.Q., Yang X.L., Zhang G., Wu S.P., Deng X.B., Xiao S.J., Liu Q.Z., Yao K.T., Xiao G.H. (2013). Nuclear Beta-Catenin Accumulation Is Associated with Increased Expression of Nanog Protein and Predicts Poor Prognosis of Non-Small Cell Lung Cancer. J. Transl. Med..

